# Designing for mild winters: evidence-based thermal comfort benchmarks from urban parks in a sub-tropical city

**DOI:** 10.1007/s00484-026-03190-9

**Published:** 2026-04-07

**Authors:** Kevin Lau, Lingye Yao, Edward Ng

**Affiliations:** 1 Department of Civil, Environmental and Natural Resources Engineering, Luleå University of Technology, Luleå, Sweden; 2 School of Architecture, The Chinese University of Hong Kong, Hong Kong, P.R. China

**Keywords:** Outdoor thermal comfort, Thermal comfort thresholds, Public space design, Sub-tropical, Ageing population

## Abstract

Outdoor thermal comfort in the cool season remains under-specified for humid subtropical cities. We conducted a winter field study across four urban parks in Hong Kong, collecting 611 paired questionnaire-microclimate records (December-March). On-site measurements (air temperature, globe temperature, humidity, wind) were linked at 60-s resolution to Cantonese surveys of thermal sensation. Neutral temperature was estimated with ordinary least squares and confirmed with proportional-odds models; 2,000-fold bootstrap confidence intervals and 0.5 °C binned regressions quantified uncertainty and sensitivity. Whole-sample neutral points were Air Temperature (T_a_) 20.6 °C (95% CI 19.7–21.6 °C), Physiological Equivalent Temperature (PET) 23.7 °C (22.2–25.8 °C), and Universal Thermal Climate Index (UTCI) 20.7 °C (19.3–22.5 °C). T_a_ was the strongest single predictor of mean thermal sensation in binned fits (R² = 0.62), followed by UTCI (R² = 0.54) and PET (R²= 0.22). Females showed slightly steeper vote-temperature slopes but similar neutralities to males. Age had a clear effect: neutral UTCI rose from 18.6 °C (< 60 years) to 23.1 °C (> 75 years), with flatter slopes in older cohorts. Park type also mattered. Neutral PET and UTCI were about 2 °C warmer in larger, greener district parks than in compact street parks, consistent with higher sky-view and lower turbulence. Robustness checks (ordinal-logit, trimmed bins, meteorological filtering) shifted whole-sample neutralities by ≤ 0.4 °C. We propose UTCI 20–22 °C as a practical winter design target for subtropical parks, complemented by 2–3 °C warmer micro-niches for older users through solar access and wind shelter. These calibrated benchmarks support climate-responsive park design that sustains outdoor use through mild winters in ageing, high-density cities.

**Supplementary Information:**

The online version contains supplementary material available at 10.1007/s00484-026-03190-9.

## Introduction

Walkable streets, podium gardens and pocket parks function as vital “third places” in high-density Asian cities, sustaining daily physical activity, casual social interaction and psychological restoration. Even outside the hot season, pronounced spatial contrasts in solar exposure, wind shelter and long-wave exchange can make one public space feel inviting while another, only metres away, deters use. A winter-round survey in Guangzhou showed that mean Physiological Equivalent Temperature (PET) still spans more than 20 °C between compact and open settings, with a neutral PET of about 20 °C for the cool season and sharply reduced stay times once PET falls below that value (Fang et al. 2019). In neighbouring Hong Kong, Lau et al. (2019) reported similarly large intra-urban gradients in mean radiant temperature and subjective thermal sensation across Local Climate Zones, confirming that urban morphology, and by extension design interventions, remains a potent lever on outdoor experience even during the milder months. Yet systematic winter-specific comfort benchmarks are sparse: most field campaigns focus on mitigating extreme summer heat, while landmark subtropical studies such as the longitudinal experiment in Hong Kong include relatively few cool-season observations and small sample sizes, limiting their generalisability (Cheng et al. 2012). Designers therefore lack evidence on how best to exploit moderate solar gains and wind shelter to encourage outdoor life during the cool season.

The knowledge gap is critical in light of East and South-East Asia’s rapid demographic transition: residents aged ≥ 60 years already constitute more than one-fifth of the population in several subtropical megacities, a share projected to double by 2050. In Hong Kong, the elderly population, specifically those aged 65 and over, is projected to reach 30% of the total population by 2040 (Census and Statistics Department, 2023). Ageing physiology, characterised by lower metabolic heat production, attenuated vasodilation and slower re-warming, narrows the behavioural thermoneutral zone, making older adults more sensitive to even modest cold stress. Controlled-environment and field studies concur that seniors require operative or equivalent temperature 1–3 °C higher than younger adults to judge conditions “neutral”, and they curtail outdoor exposure more sharply once discomfort sets in (Chen et al. 2018; Leng et al. 2020). Robust, age- and sex-differentiated winter comfort data are therefore indispensable if public space design is to remain inclusive as cities age.

Over the past decade the literature on outdoor human-biometeorology has begun to disaggregate results by age, recognising that seniors differ from younger adults in both physiological heat exchange and behavioural adaptation. Survey measurement campaigns in age-friendly housing estates in Shanghai found that the elderly already report thermal discomfort at PET values that younger residents still rate as neutral, and adopt adaptive actions such as seeking sun, adding layers at lower radiant loads than other cohorts (Li et al. 2025a, 2025b). Controlled climatic-chamber work confirms that reduced sweating efficiency and slower vasodilation underlie this heightened sensitivity, prompting calls for age-specific comfort standards (Xu et al. 2025). More recently, multi season field surveys in Chongqing’s hot and humid basin showed statistically significant upward shifts of about 2 °C in elderly neutral PET and Universal Thermal Climate Index (UTCI) in both summer and winter, with the gap between seasons narrowing in the older cohort, which is evidence that ageing amplifies cold discomfort as well as heat stress (Qin et al. 2025). Complementary results from an autumn campaign in a cold-region urban park indicate that preferred PET and acceptable ranges are systematically warmer for seniors, and that daylight-weighted mean radiant temperature is the single strongest predictor of their thermal appraisal (Hu et al. 2025). Despite this progress, most studies target specialised facilities such as nursing homes or sheltered courtyards, leaving ordinary public spaces under-represented. Cool-season data are also sparse that few studies with questionnaires involving seniors were collected in meteorological winter.

For humid-subtropical megacities the evidence base is even thinner. A cross-season study in Guangzhou reported neutral PET values of 19–25 °C but pooled late autumn and winter data, masking true mid-winter expectations (Fang et al. 2019). A follow-up analysis from the same region showed that thermal sensitivity, defined as the slope of Thermal Sensation Vote (TSV) versus PET, varies systematically with season, but winter records accounted for only 18% of the dataset, resulting in wide confidence bands (Feng et al. 2021). Outside China, a winter-focused investigation of blue-green infrastructure in Isfahan (cold-arid climate) demonstrated that PET in vegetated spaces can remain 3–4 °C cooler than in paved areas even in January; however, aridity limits generalisation to humid zones (Dashti et al. 2024). A subtropical, coastal case study from Sari, Iran used RayMan simulations to map comfort windows but relied solely on long-term weather-station data with no parallel subjective survey, precluding calibration of neutral points or acceptability bands (Abdollahzadeh and Biloria 2021). Consequently, large-sample, winter-exclusive comfort benchmarks for high-density humid subtropical climate are still lacking while sex- and age-related shifts in these benchmarks are yet quantified. Addressing these gaps is essential for planners who wish not merely to mitigate summer heat but to harness moderate winter sunshine and wind shelter to extend the usable outdoor season for rapidly ageing urban populations.

While summer heat stress has been documented extensively in East Asian megacities, comfort benchmarks in winter season remain far less certain, particularly with respect to how neutral and acceptable temperature ranges shift across demographic groups. This study therefore aims to.

- Derive winter-specific neutral and acceptable temperature ranges for three commonly used thermal indices, namely air temperature, PET and UTCI, based on 611 paired micro-meteorological and questionnaire records collected in Hong Kong public open spaces; and.
- Quantify demographic differences in those neutral ranges by sex (male, female, other/unknown) and by three age bands (< 60, 60–75, > 75 years).

By establishing statistically robust cool-season comfort thresholds and highlighting demographic shifts that are meaningful for design, the study offers actionable guidance for climate-responsive planning of public outdoor spaces, for example, sizing sun-exposed seating zones, orienting wind screens and scheduling age-friendly outdoor programmes during the cool months.

## Materials and methods

### Survey sites

Hong Kong (22°17′N, 114°09′E) has a humid subtropical climate (Köppen Cwa) dominated in winter by the East Asian northerly monsoon. Long term records from the Hong Kong Observatory show that January, the climatological middle of winter, has a mean air temperature of 16.2 °C for the period from 1991 to 2020 (daily range of 14 to 18 °C), a mean relative humidity of 74%, and the lowest annual precipitation total (about 32 mm) under predominantly clear skies (Hong Kong Observatory, 2024). Despite the mild averages, cold-surge outbreaks associated with the Siberian High can drive urban minima below 10 °C several times per season and reinforce north-easterly surface winds that frequently exceed 6 ms⁻¹ (Wu and Leung 2009). Recent climatological analyses further show a statistically significant warming trend of + 0.28 °C per decade in winter minima, superimposed on the monsoon-driven variability (Chan et al., 2012). These characteristics make Hong Kong a pertinent testbed for winter outdoor comfort research in rapidly warming yet still seasonally cool subtropical cities.

**Fig. 1:**
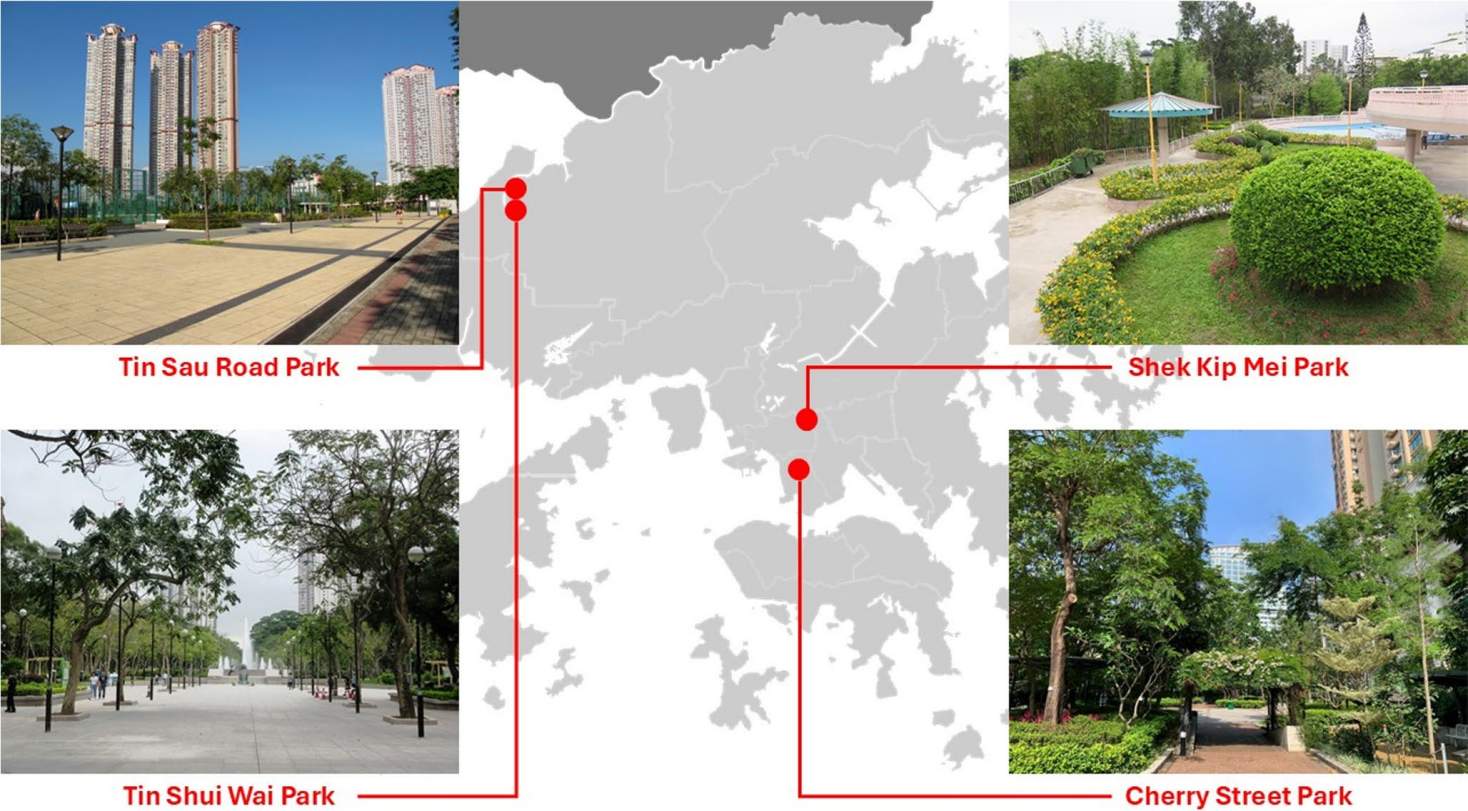
Location of the survey sites in Hong Kong (Source: LCSD)

Table 1 lists the four parks selected for the campaign. Under the Leisure and Cultural Services Department (LCSD) hierarchy two are District Parks, serving large catchments with multi-hectare footprints, while the other two are smaller Street Parks embedded in their neighbourhood fabric (Fig. 1). Together they span a useful gradient of sky view factor, wind exposure and shading that shapes winter microclimates in Hong Kong. These four parks thus provide contrasting combinations of sunshine, shading and ventilation conditions against which to examine winter outdoor thermal perception.

**Table 1:** Survey sites: classification, size and key micro-environmental attributes of the four Hong Kong urban parks investigated in the survey campaign

Park (ID)	Key micro-environmental attributes	Size & context
Tin Shui Wai Park (P-1)	Broad artificial lake and open lawns provide the highest sky-view factor; bordered by 30- to 40-storey residential blocks that funnel northerly winter breezes across the water surface.	≈ 14.9 ha in Tin Shui Wai New Town, Yuen Long District
Shek Kip Mei Park (P-2)	Terraced topography on a south-facing hillside with mature tree belts affords substantial winter shade and localised wind shelter; open sports pitches occupy the central plateau.	≈ 7.9 ha in Sham Shui Po District
Cherry Street Park (P-3)	Reclamation-edge green with open piazza and jogging loop; flanked by elevated highway decks that admit full solar exposure at midday but channel east-west winds.	≈ 3.8 ha in Tai Kok Tsui
Tin Sau Road Park (P-4)	Compact neighbourhood park adjacent to light-rail stop; mix of hard-court sports facilities and open lawn, moderate sky-view factor but relatively windy due to proximity to fishpond flood-plain.	≈ 1.2 ha in Tin Shui Wai North

### Protocol of the thermal comfort survey

The survey campaign ran from 3 December 2024 to 18 March 2025, a span that covers the entire cool, dry winter and the early spring transition. Field teams worked on selected days between 08:00 and 18:00 local time in the four study parks to sample the diurnal thermal range. Within each park, surveys were administered using an on-site intercept approach that deliberately covered common activity nodes and micro-environmental settings. Interview teams rotated between (i) sun-exposed open areas (e.g., lawns, waterfront edges, open plazas), (ii) shaded or semi-shaded areas under tree belts or pergola structures, and (iii) zones adjacent to frequently used park facilities such as seating clusters, walking loops, and exercise areas. This rotation was intended to sample the range of winter thermal exposures experienced by typical park users rather than focusing on a single micro-site condition.

**Fig. 2:**
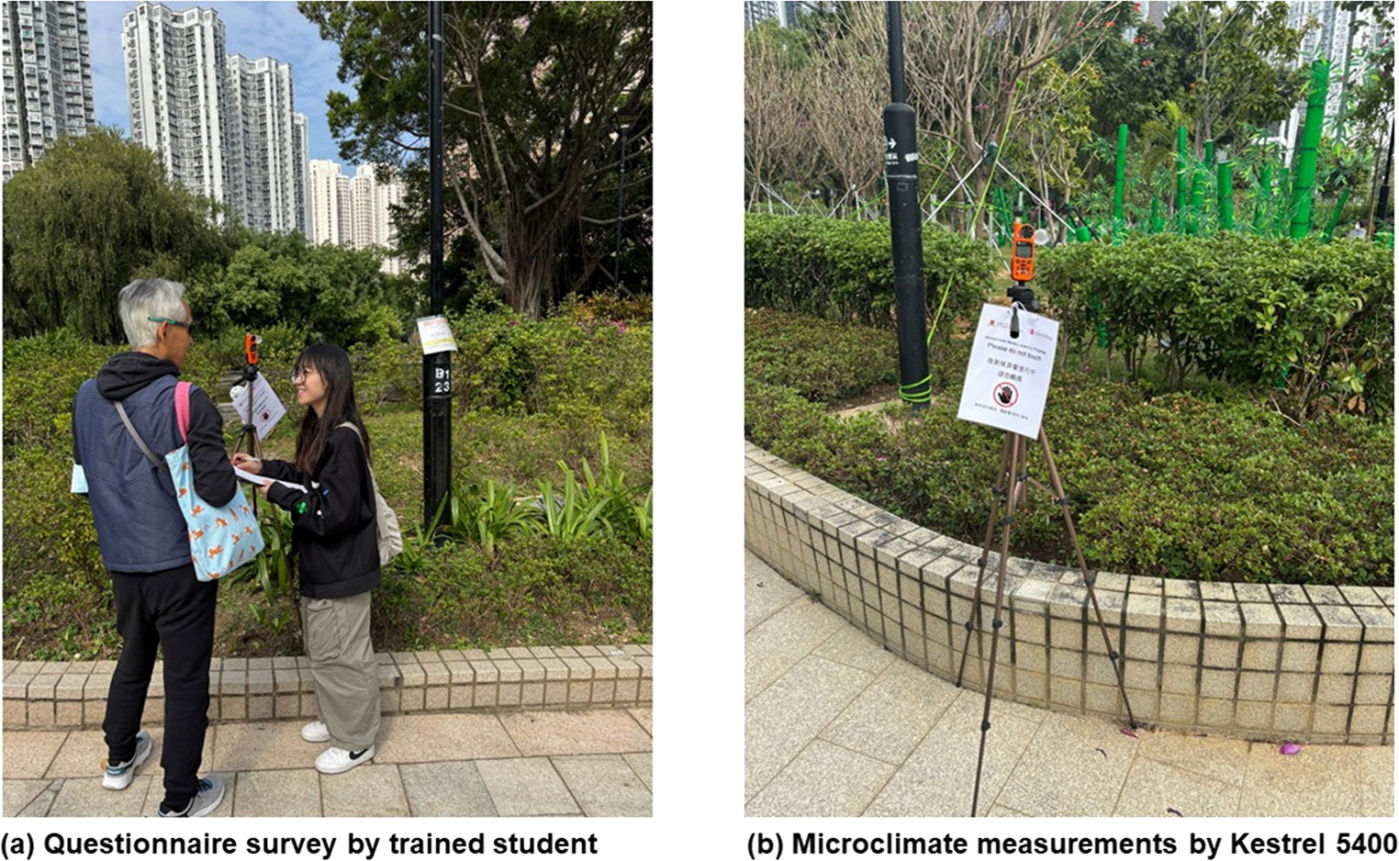
Field campaign: (**a**) Questionnaire survey, (**b**) Microclimatic measurement

We applied an on-site intercept convenience sampling strategy targeting current park users. Interviewers approached adults who were seated, strolling, or undertaking light exercise, prioritising individuals who had remained outdoors for at least 15 min to ensure sufficient exposure to local conditions. To maintain a direct link between subjective votes and microclimatic observations, recruitment was restricted to individuals located within approximately 10 m of the mobile measurement station at the time of approach (Fig. 2a). This non-probability approach, widely used in outdoor-comfort fieldwork, yields a user-centred snapshot of thermal perception under real exposure conditions. After eligibility screening (participants were aged ≥ 18 years, had remained outdoors for ≥ 15 min, and were not engaged in vigorous sport, thereby focusing the sample on sedentary to light-intensity winter park use) and subsequent data cleaning (sensor or survey omissions), 611 paired survey-measurement records out of 621 completed interviews remained for analysis. Activity in the preceding 15 min was recorded for each respondent and converted to metabolic rate (met) for PET calculations, while UTCI was interpreted primarily as an environmental exposure index.

The questionnaire was delivered exclusively in Cantonese, following the wording, flow and variable coding of two validated studies of outdoor thermal comfort (Lau et al. 2021; Lau and Choi 2021). Key questionnaire items retained or adapted from the two reference studies included: (i) thermal appraisal, comprising the 7-point ASHRAE Thermal Sensation Vote (TSV, −3 = cold to + 3 = hot), a single overall-comfort item, and a preferred-temperature change question; (ii) contextual variables, namely current activity and a clothing checklist subsequently converted to clo values; and (iii) demographics, recording exact age (later recoded into < 60, 60–75, > 75 years) and sex (1 = male, 2 = female, 3 = other/unknown). The questionnaire took approximately 5 min per respondents.

Immediately on completing the interview, the interviewer noted the starting and finishing time which linked to the micro-meteorological record within a buffer of 30 s. Records falling outside that tolerance or lacking a TSV were discarded during quality control. This protocol ensures that every subjective vote is traceable to a high-resolution, co-located microclimatic observation while capturing the spontaneous, real-world experiences of winter park users.

### Micro-climatic measurements

All microclimatic variables were recorded with a Kestrel 5400 Heat Stress Tracker (Nielsen-Kellerman, USA) that integrates a 25 mm black-copper globe, fast-response thermistor (Fig. 2b), capacitive humidity sensor and impeller anemometer in a naturally ventilated housing. The instrument was selected because it enables a compact, tripod-mounted and time-synchronised mobile station suitable for intercept thermal comfort surveys, where co-located, high-frequency measurements are required at the same position and time as each questionnaire response. The unit was mounted on a carbon-fibre tripod at 1.1 m above ground level, i.e. the average height of the human centre of thermal mass in a standing posture recommended by ISO 7726. The stated sensor accuracies (air temperature (T_a_) ± 0.3 °C, globe temperature (T_g_) ± 0.5 °C, relative humidity (RH) ± 2%, wind speed (WS) ± 3%) are consistent with measurement performance commonly adopted in outdoor thermal comfort field campaigns, and the deployment followed standard guidance for sensor height and globe-based estimation of mean radiant temperature. Data were logged at 60-s intervals, time-stamped via the Kestrel LiNK interface and synchronised automatically with the tablet that hosted the questionnaire.

To verify measurement consistency, the Kestrel was co-located with a Testo 440 temperature-humidity probe and its companion hot-wire air-flow sensor before and after the campaign at the same height (1.1 m) and over matched time windows under representative winter conditions. Agreement was evaluated by comparing concurrent readings for T_a_, RH and WS, and discrepancies remained within the manufacturers’ stated tolerances. Mean radiant temperature (T_mrt_) was derived from globe temperature using the ISO 7726 globe method, which uses T_g_, T_a_ and WS together with globe geometry and emissivity. Since globe-based T_mrt_ estimates are more sensitive to wind speed and radiant forcing than direct T_a_ measurements, we interpret PET and UTCI as composite indices subject to propagated measurement uncertainty, and we therefore retain T_a_ as a parallel benchmark in the TSV analyses. PET and UTCI were then computed using pythermalcomfort v2.5 based on the measured T_a_, RH, WS and derived T_mrt_ together with respondent-specific clothing insulation and activity inputs. Clothing insulation was obtained from the clothing checklist and converted to clo using ISO 9920, and activity was coded to a corresponding metabolic rate based on the reported activity category.

The Kestrel 5400 is widely used in outdoor-comfort research, having supported studies on (i) sky view factor and greening effects on pedestrian comfort in Shanghai (Liu et al. 2024), (ii) micro- and macro-scale heat-island mapping in Bradford, United Kingdom (Templeton and Taleghani 2024), (iii) multi-season thermal perception surveys in Hong Kong public housing estates (Li et al. 2025a, 2025b), and (iv) optimisation for courtyard design in hot-arid Tehran (Azimi and Shafaat 2024). Adopting the same instrument therefore facilitates cross-study comparability while ensuring that the high temporal resolution and integrated globe sensor needed for winter comfort analysis are met.

### Data processing, statistical analysis and quality assurance

A total of 621 questionnaires were completed during the field campaign. Each questionnaire record was then time-matched to the nearest 60-s microclimatic log within a ± 30 s tolerance. Ten records could not be paired because the corresponding microclimatic measurements were missing or incomplete within the matching window. The resulting paired analytic dataset comprised 611 questionnaire-measurement records, and this paired sample forms the basis for all regression, neutral-value estimation, and subgroup analyses unless otherwise stated.

Clothing insulation and activity were treated as individual-level inputs. Clothing insulation was derived from the clothing checklist by assigning each reported garment an ISO 9920 reference insulation value and then combining garments into a total ensemble insulation using an additive approach (Table S1). In practice, the base layer, mid layer, and outer layer items selected by the respondent were summed to obtain an ensemble clo value for that observation; accessories (e.g., hat, scarf, gloves) were added where applicable following ISO 9920 guidance. Activity was recorded using a small set of questionnaire categories describing what the respondent had been doing during the preceding 15 min (sitting/resting, standing, walking/strolling, light exercise). These categories were converted to metabolic rate (met) using standard values from established activity compendia and thermal comfort practice, and the resulting met value was applied to the corresponding respondent record (Table S2).These respondent-specific clo and metabolic inputs were used together with T_a_, RH, WS and T_mrt_ to compute PET and UTCI in pythermalcomfort v2.5, ensuring that inter-individual differences in clothing and activity were reflected in the thermal indices. This workflow avoids reliance on fixed clothing or activity assumptions and ensures that reported thermal indices reflect both environmental exposure and behavioural context.

To support graphical interpretation under heavy over-plotting and to summarise the mean TSV response across the observed exposure range, thermal metric values were additionally grouped into 0.5 °C bins and the mean TSV was computed for each bin. This binning was used only as a descriptive smoothing step for visualisation and to provide an intuitive representation of the average response pattern. All primary inference, including estimation of neutral values, subgroup contrasts, and uncertainty quantification, is based on individual-level models fitted to the full dataset. Accordingly, any goodness-of-fit statistics computed on binned means reflect fit at the bin level rather than predictive performance at the individual level.

Neutral temperature for each index was first estimated by ordinary least-squares regression of TSV on the metric. Precision was gauged with 2000-fold bootstrap confidence intervals that make no distributional assumptions and are robust to heteroscedasticity. Because TSV is ordinal, an ordinal logit model (proportional-odds) provided a sensitivity check, defining neutral as the metric value where the expected TSV equals zero. Subgroup effects were explored in separate models for sex (male, female, other/unknown) and age bands (< 60 years, 60–75 years, > 75 years); non-overlapping 95% CIs were taken to indicate a meaningful demographic shift. All analyses were run in R 4.3.2 with the tidyverse, ordinal and boot packages; figures were produced with ggplot2.

Sensor calibration was verified pre- and post-campaign by side-by-side tests with a Testo 440 temperature-humidity probe and hot-wire air flow sensor, yielding deviations within manufacturer tolerances. Residual radiant-shield bias was minimised by mounting the Kestrel 5400 on a matte-black tripod and conducting shade audits at each location. Limitations include the single-season scope, the on-site intercept convenience sampling (which favours current park users over the thermally deterred), and the potential Hawthorne effect of interviewer presence. Nevertheless, the large sample size, rigorous time-matching, bootstrap inference and cross-validated ordinal models together provide a robust basis for winter comfort benchmarks and their demographic variability. We acknowledge that uncertainty in globe-derived T_mrt_ can be higher under low wind and strong sun, and we therefore triangulate comfort patterns across T_a_ and composite indices and interpret subgroup differences in conjunction with the reported confidence intervals and robustness checks.

## Results

### Overview of the respondents

Following time-matching and quality-control screening, 611 paired questionnaire-measurement records remained for analysis (Table 2). Although 621 questionnaires were completed, 10 records could not be paired with valid simultaneous microclimatic measurements and were therefore excluded from the analytic sample. The cohort was 52.8% female and 46.5% male, with a small “other/unknown” category (0.6%). Age distribution skewed older by design: 45.2% were 60–75 years and 29.5% were above 75 years, leaving 25.3% in the adult (< 60 years) band. Overall, recent behaviour remained low intensity but spanned both static and light dynamic use. In the 15-minute pre-survey window, static activities comprised sitting/resting (28.3%) and standing (12.9%), while dynamic activities comprised walking/strolling (20.0%) and light exercise (38.6%). Aggregated to these two classes, static use accounted for 41.2% of observations and dynamic use accounted for 58.6%, indicating that the dataset reflects typical winter park behaviours dominated by sedentary to light-intensity activity rather than vigorous sport. Spatially, interviews were split between Street Parks (Cherry Street Park, Tin Sau Road Park − 34.5%) and District Parks (Tin Shui Wai Park, Shek Kip Mei Park − 65.5%). This demographically diverse, winter exclusive dataset therefore provides a robust foundation for establishing neutral temperature benchmarks and for examining sex- and age-related shifts in outdoor thermal perception.

**Table 2:** Characteristics of respondents (*n* = 611)

Sex	*n*	%		15-min activity	*n*	%
Male	289	46.5		Sitting/resting	176	28.3
Female	318	52.8		Walking/strolling	124	20.0
Other/unknown	4	0.6		Standing	80	12.9
				Light exercise	231	38.6
**Age group**				**Park type**		
< 60 yr	157	25.3		Street Park	214	34.5
60–75 yr	275	45.2		District Park	397	65.5
> 75 yr	179	29.5				

### Winter microclimatic conditions

Across the 611 matched survey records, T_a_ ranged from 11.8 °C to 28.3 °C (mean ≈ 19.0 °C), while mean radiant temperature (T_mrt_) spanned a much wider 11.5–56.8 °C, reflecting strong modulation by solar exposure in the open-sky winter environment. T_g_ and derived indices followed suit, yielding PET values of 7.9–35.6 °C (mean ≈ 21.3 °C) and UTCI values of −0.1–30.2 °C (mean ≈ 17.9 °C). RH averaged 49% but dropped below 30% on several clear, dry-monsoon days. WS measured at 1.1 m were light overall (median = 0.50 m s⁻¹), yet maxima exceeded 1.5 m s⁻¹ during northeasterly-surge events, the conditions felt most keenly in the street parks. Descriptive statistics for all variables are presented in Table 3.

**Table 3:** Descriptive statistics of winter microclimatic variables (*n* = 611)

	*n*	Mean	SD	Min	Q1	Median	Q3	Max
T_a_ (°C)	611	18.96	3.46	11.8	16.34	19.01	22.05	28.28
T_g_ (°C)	611	22.82	5.27	11.72	18.21	23.39	26.13	35.62
RH (%)	611	48.95	14.61	27.73	36.19	44.63	64.21	78.93
WS (m s^− 1^)	611	0.52	0.29	0	0.31	0.5	0.7	1.54
T_mrt_ (°C)	611	28.72	9.47	11.5	21.15	27.33	34.48	56.75
PET (°C)	611	20.92	5.75	8.54	16.24	21.85	24.75	35.17
UTCI (°C)	611	18.04	5.57	0.91	13.89	18.88	22.55	30.15

### Relationship between thermal sensation and temperature metrics

**Fig. 3:**
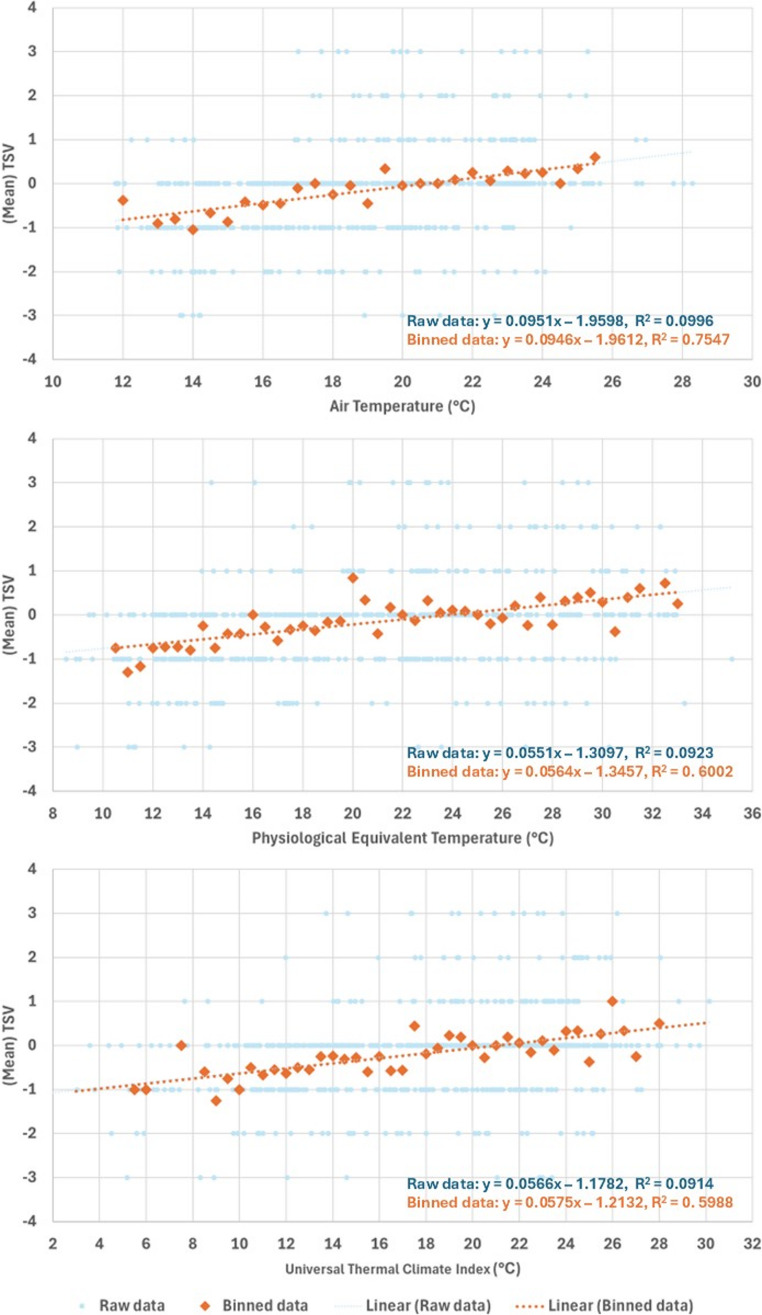
Scatterplots of thermal sensation vote vs three temperature metrics (T_a_, PET, UTCI) for both raw and binned data

Figure 3 presents scatterplots of individual TSV plotted against T_a_, PET, and UTCI, respectively. Each plot includes raw TSV data (light scatter), 0.5 °C binned means (black dots), and a fitted linear regression line. Table 4 presents the binned linear regression equations and R² values for the relationship between TSV and three thermal indices (T_a_, PET, and UTCI) across the whole sample and key subgroups defined by sex, age, and park type. The slope of each equation represents the degree of thermal sensitivity, i.e. the change in TSV per 1 °C increase in the thermal metric. Higher slope values indicate greater responsiveness to changes in environmental conditions, while the R² values reflect how consistently the subgroup reacts to thermal stimuli.

**Table 4:** Binned regression equations and R² values (raw and binned) for TSV vs. thermal indices by subgroup

Group	*n*	Metric	Equation (binned)	*R*² raw	*R*² binned
Whole sample	611	T_a_	−1.47 + 0.07x	0.10	0.62
		PET	−0.99 + 0.04x	0.09	0.22
		UTCI	−1.20 + 0.06x	0.10	0.54
Male	289	T_a_	−1.55 + 0.07x	0.07	0.55
		PET	−1.12 + 0.05x	0.09	0.22
		UTCI	−1.07 + 0.05x	0.07	0.50
Female	318	T_a_	−1.40 + 0.07x	0.15	0.67
		PET	−1.22 + 0.05x	0.11	0.24
		UTCI	−1.42 + 0.07x	0.13	0.55
Age < 60 yr	157	T_a_	−1.87 + 0.09x	0.07	0.68
		PET	−1.39 + 0.06x	0.06	0.33
		UTCI	−1.03 + 0.05x	0.07	0.34
Age 60–75 yr	275	T_a_	−1.51 + 0.07x	0.11	0.53
		PET	−1.08 + 0.04x	0.10	0.20
		UTCI	−1.03 + 0.05x	0.10	0.29
Age > 75 yr	179	T_a_	−1.67 + 0.07x	0.05	0.44
		PET	−1.49 + 0.05x	0.03	0.26
		UTCI	−1.40 + 0.06x	0.06	0.39
Street parks	214	T_a_	−0.88 + 0.05x	0.09	0.58
		PET	−0.81 + 0.03x	0.08	0.13
		UTCI	−0.79 + 0.04x	0.07	0.38
District parks	397	T_a_	−2.31 + 0.11x	0.12	0.47
		PET	−1.60 + 0.05x	0.10	0.27
		UTCI	−1.94 + 0.08x	0.15	0.56

Across all three thermal metrics, TSV increases with warmer conditions, but the individual-level relationships are modest, with raw R² values around 0.09 to 0.10. This is expected in outdoor comfort field surveys where inter-individual differences in physiology, clothing, activity, thermal history, and micro-site variation introduce substantial variance at any given exposure. To reduce over-plotting and to visualise the mean response more clearly, we additionally plotted 0.5 °C binned mean TSV values. As anticipated, regressions on binned means yield higher R² values because averaging reduces within-bin dispersion. These binned R² values therefore describe how closely the mean TSV trend follows each thermal metric at the aggregated level, and they should not be interpreted as individual-level predictive power. Within this descriptive framing, air temperature shows the tightest association with mean TSV in the pooled winter dataset. However, this should be interpreted as a dataset-specific empirical pattern rather than evidence that air temperature is inherently a superior physiological descriptor. PET and UTCI embed additional drivers of thermal perception, particularly radiation and wind effects, which can peak earlier in the day than air temperature under clear winter skies and may therefore exhibit a different diurnal phase. Consistent with this interpretation, index performance varies by context in our data: in the larger and more exposed district parks, UTCI captures wind and radiant influences more effectively and shows comparable coherence to air temperature when responses are aggregated. 

Table 4 summarizes the binned equations and R^2^ values for thermal sensation vote vs. three temperature metrics, grouped by sex, age, and park type. Clear sex-related differences emerged. Among male respondents, thermal sensitivity is moderate: the T_a_ slope is 0.07 with a binned R² of 0.55, while PET and UTCI yield slopes of 0.049 and 0.050, respectively. Female respondents, by contrast, are more responsive across all indices. Their TSV increases by 0.072 TSV °C⁻¹ with T_a_ and 0.069 with UTCI. Moreover, they showed notably higher explanatory power. R² values for females reach 0.67 for T_a_ and 0.55 for UTCI, surpassing those of males in every index. These findings suggest that female park users perceived and reported temperature changes more sensitively and consistently, particularly in response to integrated indices like UTCI.

Age related variation in thermal sensitivity is even more pronounced. The youngest group (< 60 years) shows the steepest slopes across all metrics: 0.090 for T_a_, 0.058 for PET, and 0.069 for UTCI. Their R² values are also the highest among age groups, 0.68 for T_a_ and more than 0.33 for both PET and UTCI, indicating strong and consistent thermal appraisal. Both slope and explanatory power decline as age increases. For individuals aged 60–75 years, the slope for T_a_ drops to 0.081, with a binned R² of 0.53. Among the oldest group (> 75 years), the slope for Ta falls further to 0.078, while PET drops to 0.042 with an R² of only 0.26. UTCI retains slightly more explanatory power (slope 0.053, R² = 0.39), likely due to its inclusion of wind-chill effects, which remain perceptually salient among older individuals. These results confirm previous findings that thermal sensitivity diminishes with age, both in magnitude and consistency (Wu et al. 2023).

Park setting also influenced TSV-metric relationships. In street parks (Cherry Street Park and Tin Sau Road Park), respondents show strong reliance on T_a_ (slope = 0.096, R² = 0.58), while PET showed weaker coherence at the binned level (slope = 0.054, R² = 0.13). As site geometry was not formally quantified, we interpret this pattern cautiously. However, it is consistent with the observed morphology described in Table 1, where the street parks are compact and embedded within transport and neighbourhood structures, which may dampen variability in radiant load relative to air temperature. In contrast, users of district parks (Tin Shui Wai and Shek Kip Mei Parks) show higher sensitivity to UTCI, with a slope of 0.082 and R² of 0.56, the highest among all subgroups. These larger, vegetated parks span a wider mix of open lawns, water edges, terraced slopes and tree belts, which may accentuate combined radiation and wind effects and thereby make UTCI comparatively more representative of the perceived thermal environment.

The regression analyses confirm that thermal sensitivity is not uniform across populations or contexts. T_a_ consistently yields the highest predictive power, but UTCI becomes more relevant in district parks and among females. Age reduces both sensitivity and vote coherence, suggesting that older individuals may require greater environmental contrasts to register thermal change. These findings carry direct implications for design: younger users may respond to subtle improvements, while older adults may benefit from more pronounced interventions such as strategically located windbreaks, sunny seating areas, or surface materials that retain warmth. Integrating these subgroup insights into climate-responsive public-space design will enhance thermal equity and support year-round use across all demographics.

### Neutral temperature and subgroup contrasts

Table 5 reports winter neutral temperature (defined by the thermal metric at which mean TSV = 0) for T_a_, PET and UTCI. Estimates are based on linear regression, with 2 000-fold bootstrap medians and 95% confidence intervals (CI). Values are presented for the whole sample and for sub-groups defined by sex, age and park type. Across all valid observations, the neutral points were 20.6 °C (95% CI 19.7–21.6 °C) for T_a_, 23.7 °C (22.2–25.8 °C) for PET, and 20.7 °C (19.3–22.5 °C) for UTCI. More than 95% of comfort votes were “acceptable” (overall comfort ≥ 0) throughout the observed range, so an 80% acceptability band could not be bounded, which is an expected outcome in Hong Kong’s mild winter.

**Table 5:** Neutral thermal indices (median and 95% CI) by subgroup

Group	*n*	T_a_ (°C)	PET (°C)	UTCI (°C)
Whole sample	611	20.6 (19.7–21.6)	23.7 (22.2–25.8)	20.7 (19.3–22.5)
Male	289	20.9 (19.5–22.9)	24.1 (21.8–27.7)	21.4 (19.6–23.8)
Female	318	20.3 (19.1–21.7)	23.3 (21.3–25.4)	20.2 (18.8–22.3)
Age < 60 yr	157	19.1 (17.3–20.8)	21.6 (17.8–24.8)	18.6 (16.4–21.4)
Age 60–75 yr	275	20.7 (19.1–22.5)	23.7 (21.2–26.9)	21.0 (19.2–23.6)
Age > 75 yr	179	22.1 (20.2–24.1)	26.0 (22.0–31.5)	23.1 (20.1–26.5)
Street parks	214	19.9 (18.6–21.1)	23.4 (21.0–25.3)	19.8 (18.1–21.9)
District parks	397	21.0 (19.4–22.6)	27.3 (24.0–30.9)	21.7 (20.2–23.8)

Females displayed slightly cooler neutral T_a_ (20.3 °C) than males (20.9 °C), but CIs overlap (19.1–21.7 °C vs. 19.5–22.9 °C), indicating no statistically clear sex gap. For UTCI the pattern was similar (20.2 °C vs. 21.4 °C). PET showed the largest difference (23.3 vs. 24.1 °C), yet again confidence intervals intersect. The modest sex effect aligns with the regression slopes in Sect. 3.3: women were marginally more sensitive but not enough to shift neutral points beyond uncertainty bounds.

Age affected neutrality more decisively. Adults < 60 year reached neutrality at the coolest values, i.e. 19.1 °C T_a_, 21.6 °C PET, 18.6 °C UTCI. Neutral points rose by ~ 2 °C for the 60–75 years group and by a further degree for the > 75 years cohort, peaking at 22.1 °C T_a_ and 26.0 °C PET. Confidence intervals show only partial overlap, confirming that older respondents prefer or require warmer winter conditions to feel thermally neutral.

Neutral temperatures were also park-type dependent. Street-park users reported neutrality at 19.8 °C UTCI and 23.4 °C PET, approximately 1–2 °C cooler than district-park users (21.7 °C UTCI; 27.3 °C PET). As we did not quantify geometric parameters such as sky view factor, enclosure ratios, or canopy cover, the physical mechanisms cannot be confirmed directly. Nonetheless, the direction of the difference is consistent with the contrasting site characteristics described in Table 1, where the district parks include broader lawns, open-water edges, and more extensive open-sky areas alongside sheltered niches, whereas the street parks are smaller and embedded within denser neighbourhood fabric. These observed contrasts plausibly modify winter radiant exposure and wind shelter and therefore may contribute to the park-type differences in thermal neutrality.

### Sensitivity and robustness checks

Several supplementary analyses were conducted to verify that the neutral-temperature benchmarks and TSV-temperature relationships reported above are not artefacts of modelling choice or data idiosyncrasies. As TSV is an ordered categorical variable, we re-estimated neutral temperature with a proportional-odds logit model. For the whole sample, the neutral points shifted by no more than 0.4 °C relative to the Ordinary Least Squares (OLS) values, namely 20.3 °C for T_a_, 23.3 °C for PET, and 20.4 °C for UTCI. Subgroup differences followed the same rank order (Age > 75 > Age 60–75 > Age < 60; district > street), confirming that the linear assumption did not bias the main results.

Bootstrap distributions were examined for skewness and multimodality. Air-temperature neutrals showed tight, symmetric dispersions (inter-quartile widths: 1.5 °C whole sample; 2 °C in sub-groups). PET and UTCI neutrals were slightly right-skewed, especially in the > 75 years cohort and district parks, reflecting a handful of sunny-calm survey periods with elevated radiant loads. Nevertheless, 95% CIs remained narrower than ± 3 °C for every index and group, indicating statistical stability.

The TSV regressions were repeated after deleting 0.5 °C bins with fewer than five votes (to dampen sparse tails) and it changed slope estimates by ≤ 0.01 TSV °C⁻¹ and binned R² by < 0.03 across all metrics and groups. Neutral temperature moved by at most 0.2 °C, demonstrating that the fitted relationships are not sensitive to low-frequency extremes. Excluding the two strongest cold-surge days (daily mean air temperature < 12 °C) and the three warmest episodes (> 26 °C) narrowed PET and UTCI ranges but shifted neutral points by less than 0.5 °C and left subgroup ordering unchanged, indicating that moderate winter days dominate the parameter estimation.

Taken together, these checks confirm that the study’s core findings, namely neutral temperature around 20 to 21 °C UTCI, a 2 °C upward shift for the oldest cohort, and higher UTCI sensitivity in district parks, are robust to the modelling framework, sampling variability, and meteorological filtering. Designers and researchers can therefore apply the reported benchmarks with confidence when formulating winter comfort strategies for humid subtropical public spaces.

## Discussion

### Winter neutral temperature in regional context

Our winter field campaign confirms that neutral outdoor conditions for Hong Kong park users cluster around 20–21 °C T_a_, 23–24 °C PET and 20–21 °C UTCI (Table 5). These values arise from more than six hundred questionnaire-instrument pairs, giving the tightest bootstrap confidence bands yet reported for a humid-subtropical metropolis. The results also reveal systematic modifiers: neutral UTCI rises by approximately 2 °C between adults < 60 years and those > 75 years, and by a similar margin between compact street parks and expansive district parks. Although air temperature remains the single strongest predictor of mean TSV (binned R² ≈ 0.6), UTCI outperforms PET and even air temperature in vegetated, wind-sheltered settings, underscoring the role of combined radiation-and-wind effects in shaping winter comfort. These outcome magnitudes provide a robust empirical baseline against which regional studies can be benchmarked.

When compared with the limited literature for cool seasons in similar climates, the Hong Kong medians fall within the published range, although toward its warmer end. In Guangzhou (23° N), field surveys that spanned late-autumn to winter reported neutral PET values of 22 °C to 24 °C depending on sky view factor, and a neutral UTCI close to 20 °C; our district-park PET of 27 °C is higher, but our city-wide median of 23.7 °C aligns with their mid-density sites (Feng et al. 2021). A more recent seasonal analysis in the same city placed winter neutral PET at 23 °C (95% CI: 21–25 °C), virtually identical to our whole-sample estimate, while confirming that winter comfort bands are markedly narrower than summer bands (Fang et al. 2019).

A recent study in Chengdu, located in the hot-summer, cold-winter zone of inland China, offers a useful benchmark. It was reported that neutral UTCI and PET are 18.2 °C and 12.8 °C respectively during the January survey, rising to 24.8 °C UTCI and 15.1 °C PET in summer (Wei et al. 2022). Compared to our findings in Hong Kong (20.7 °C UTCI, 23.7 °C PET), UTCI neutrality is about 2 °C cooler, while PET neutrality is nearly 11 °C lower in Chengdu. This large PET difference reflects Chengdu’s heavily shaded, cloud-prone winter conditions (SVF ≈ 0.05 in some plots), which suppress mean radiant temperature. In contrast, Hong Kong’s clearer winter skies allow strong solar gains, elevating T_mrt_ and PET even when air temperature is mild. These results confirm that PET neutrality varies strongly with radiant load, while UTCI remains relatively stable across subtropical sites with similar wind exposure. Across all these studies, PET and UTCI neutrals derived from ordinal-logit models differ from simple OLS fits by less than 0.5 °C, mirroring the robustness checks and suggesting methodological consistency across regions.

A further interpretive consideration is the diurnal phase difference between air temperature and radiation driven indices in clear winter weather. In humid subtropical winters, shortwave radiation and mean radiant temperature commonly peak around solar noon, whereas air temperature often peaks later in the afternoon due to surface and atmospheric thermal inertia. As a result, PET and UTCI can reach their daily maxima earlier than air temperature under the same day, even when all variables are measured simultaneously at the time of the interview. This timing difference does not affect our regression analysis, which uses the instantaneous microclimate observed concurrently with each TSV. It does, however, caution against treating air temperature as a complete proxy for the thermal experience across the diurnal cycle, particularly when the design objective is to capture midday solar warmth or wind exposure conditions that are better represented by mean radiant temperature and wind sensitive indices.

Our finding that age elevates neutral temperature by up to 3 °C is broadly consistent with controlled-chamber work showing reduced vasodilatory capacity in seniors (Greaney et al. 2021), but contrasts with a Shanghai nursing-home study where the old-old cohort displayed only a 1 °C PET rise relative to younger residents (Qin et al. 2025). Two factors may explain the larger gap observed here. First, our sample includes healthy community dwelling elders who engage in light exercise, exposing them to a wider operative-temperature range than frail residents restricted to sheltered courtyards. Second, the maritime winter in Hong Kong is breezier than inland Shanghai’s, amplifying convective heat loss and perhaps heightening the old-old desire for radiant warmth. The practical implication is that designers should supply wind-sheltered, sun-exposed niches capable of adding at least 2 °C UTCI compared with the park mean, especially in zones frequented by seniors.

Sex differences proved more nuanced. Although female respondents exhibited slightly steeper TSV slopes, neutral temperature differed by less than 0.6 °C and their confidence intervals overlapped those of males. This echoes findings from a multi-city European survey where physiological sex effects emerged mainly under heat-stress conditions, with cool-season neutral points converging across sexes (Potchter et al. 2018). Thus, winter comfort standards need not be sex-differentiated, but higher female sensitivity suggests that even minor micro-climatic improvements, e.g. blocking a 1 m s⁻¹ wind, could yield perceptible comfort gains.

Spatial context exerted a stronger influence than sex. District parks produced a warm-shift of approximately 2 °C in neutral PET and UTCI relative to street parks, mirroring earlier observations that larger sky-view factors and lower turbulence intensify shortwave and longwave radiant exchange (Chen et al. 2012). Because most Hong Kong pocket parks resemble the latter category, city-wide comfort strategies should prioritise radiant access and wind mitigation in smaller spaces, while in district parks designers might exploit the buffering capacity of canopy and grass to offer both cooler and warmer micro-niches.

Finally, our narrow bootstrap confidence intervals (< ± 3 °C for every subgroup) improve upon those reported in prior work, where sample sizes rarely exceed 300 and seasonal mixing inflates variance. By isolating meteorological winter and deploying both KDE smoothing and bin-based regression, the present study reduces estimate noise and provides a high-precision dataset for future meta-analyses. Such methodological rigour answers calls in recent review papers urging larger samples and more robust statistics in outdoor comfort research (Zheng et al. 2025). Collectively, the convergence of neutral benchmarks across Hong Kong, Guangzhou, and Chengdu, despite differing urban morphologies, suggests that a UTCI target of 20 to 22 °C and a PET target of 22 to 24 °C are broadly transferable comfort design goals for humid subtropical winters, provided designers account for the demographic and site-specific modifiers documented here.

### Demographic and site-based variation in thermal neutrality

Our dataset shows that women were only marginally cooler than men at neutrality (UTCI about 0.3 °C lower) yet displayed steeper TSV-temperature slopes, which mirrors field evidence from both warm humid and cold humid cities. On pedestrian streets in Harbin (Dfa climate) females reported higher thermal-stress votes than males under identical meteorological exposure, with a regression slope 12% steeper for air temperature (Jin et al. 2020). Taiwanese questionnaire surveys likewise found that women were less tolerant of heat and more sensitive to solar exposure than men in hot humid urban squares (Tung et al. 2014), which was attributed to different behavioural expectations rather than purely physiological causes. A Finnish study also suggested that females, on average, have higher thermal-sensitivity coefficients even when clothing and activity are held constant (Karjalainen 2007), while a recent cross-climatic synthesis showed that the sex gap enlarges in cool and transitional seasons, consistent with our winter findings (Mountzouris et al. 2025). These studies support the conclusion that micro-scale interventions such as wind shields or radiant patches will be perceived more acutely by female users, even though design set-points need not differ by sex.

Age exerted a stronger and more systematic influence. Our neutral UTCI rose from 18.6 °C (< 60 years) to 23.1 °C (> 75 years), with an equally monotonic decline in TSV-temperature slopes. A winter experiment in Chongqing community parks reported a comparable 4 °C PET rise between young adults and seniors and linked the effect to slower metabolic heat production and conservative clothing choices among the elderly (Qin et al. 2025). A study in nursing homes during summer in the continental Mediterranean climate found that older adults are less sensitive to temperature changes and have a wider comfort zone than younger adults (Baquero and Forcada 2022). A previous literature review corroborates these field results that ageing is associated with a progressive decrease in thermal perception, as revealed by increased thermal detection thresholds in the elderly (Guergova and Dufour 2011), providing a physiological basis for elevated comfort targets of approximately 2 °C UTCI in age-friendly park zones.

Spatial settings modulated comfort by an order comparable to age. Users in our district parks, characterised by large lawns with open sky, showed neutral PET about 2 °C warmer than visitors in compact street parks, reflecting higher solar gain and reduced turbulence. (Kong et al. 2021) reported up to a 19.7 °C PET reduction between sunny locations and shaded areas provided by pergolas and trees with high crown density in Suwon, while (Bai et al. 2024) reported consistently lower daytime temperature and reduced diurnal temperature ranges in urban greenspaces compared to impervious surfaces across all seasons in Shenzhen. ENVI-met modelling in Tehran demonstrated that pine trees provided the greatest daytime thermal comfort (maximum PET reduction of 2.7 °C), shrubs created higher daytime discomfort but cooler nights (Karimi et al., 2020), and greening design in Munich with trees placed in the sunlit areas of the square provided 5.2% higher cooling effect compared to the existing greening design (Zölch et al. 2019). Such convergent evidence confirms that park morphology, including sky view factor, vegetation density and wind exposure, can readily shift winter neutrality by approximately 2 °C, endorsing the strategy of providing thermally diverse “sun-pockets” and “breeze-lanes” within a single public space to satisfy heterogeneous user groups.

### Spatial and design implications for winter park environments

Our core finding of neutral UTCI of 20–21 °C (≈ 21 °C shaded air temperature) provides a clear baseline for winter park planning in humid-subtropical cities. As demonstrated in earlier section, neutral points shift upward by roughly 2–3 °C among older adults (> 75 years), indicating that seniors require warmer microclimates to maintain thermal neutrality. Similar age-driven warm shifts have been documented in Huangshan, where neutral PET among elderly park users demonstrated higher neutral PET than younger adults (Wang et al. 2023). Johansson et al. (2014) called for urban designs that offer a range of thermal conditions to accommodate demographic variability. Ma et al. (2021) suggested that by tailoring park microclimates and amenities to senior users’ needs, winter park attendance and usage can be meaningfully increased, especially when age-appropriate exercise infrastructure is combined with thermally comfortable resting areas.

The neutrality benchmarks reported here should be interpreted in the context of the activity profile captured by the survey, which is dominated by sedentary to light-intensity behaviours typical of winter park use, including resting, socialising, strolling, and low-intensity exercise. For such use, neutral UTCI around 20 to 21 °C provides a practical design target for zones intended for sitting and lingering, particularly for older users whose neutral points are warmer. At higher metabolic rates, the same meteorological conditions will be perceived as warmer, and comfort targets will shift toward cooler exposures. Rather than relying on a single ‘one-size-fits-all’ set-point, the design implication is to provide a mosaic of microclimates within the same park, including sun-exposed, wind-sheltered ‘comfort pockets’ that support static or older users, and more ventilated ‘movement corridors’ along paths and exercise loops that better accommodate walkers and more active users. In this way, neutrality temperatures derived largely from low-intensity behaviours remain highly actionable for design, while thermal diversity ensures inclusivity across a broader range of user behaviours.

Maximising solar access and maintaining high sky-view factors are among the most effective measures for raising radiant warmth in winter. In Egypt, Shata et al. (2021) demonstrated that open plaza with sky view factor > 0.7 produced UTCI about 3 °C warmer than adjacent tree-lined corridors. Similarly, Kecman et al. (2025) found that Belgrade’s urban green space exhibited PET 4 °C higher than compact street canyons, owing to increased short-wave irradiance. Conversely, wind management is crucial in narrow street parks, where winter monsoon breezes can dominate perceived temperature. Abdollahzadeh and Biloria (2021) used ENVI-met simulations in Sydney to show that low hedges and louvred screens can reduce mean wind speeds by 40%, translating into UTCI gains of 1–2 °C. It was similarly reported that wind-break planting in Phoenix pocket plazas delivered > 2 °C PET improvement (Unal and Middel 2025). Vegetation configuration must thus balance radiation and ventilation: deciduous canopy retains winter sun while providing summer shade, and understorey shrubs deflect wind without blocking beneficial low-angle radiation.

Different park activities demand distinct thermal settings. Younger or active users (walking, tai-chi) tolerate cooler, ventilated “breeze lanes,” whereas sedentary or elderly users require “sun pockets” offering + 2–3 °C UTCI. In cold northern Chinese cities, winter park attendance increased markedly in sunlit open spaces, and thermal comfort was strongly linked to microclimatic adjustments, particularly greater solar exposure that encouraged outdoor activity around midday, underscoring the value of design strategies such as shelterbelts, careful placement of deciduous trees, and targeted wind protection to optimize the use of residential open spaces in winter (Mi et al. 2020). Retrofits in existing street parks can employ modular pergolas with retractable transparent awnings (Abdollahzadeh and Biloria 2021) or movable planter arrays (Unal and Middel 2025) to dynamically tune wind flow and solar access throughout the season. Lastly, water features, while cooling in summer, should be deactivated or relocated away from senior zones in winter to avoid evaporative chill. By integrating these activity- and demographic-specific adaptations, mild-winter parks can sustain year-round liveability for all user groups.

### Methodological contributions, robustness, and limitations

This study contributes methodologically to the outdoor thermal comfort literature by combining several statistical and fieldwork refinements that together enhance the reliability and comparability of winter thermal comfort benchmarks. First, we paired high-frequency microclimatic data (1-min logging) with real-time survey timestamps, ensuring tight temporal alignment between subjective and objective records. This improves accuracy over fixed-station approaches, particularly under rapidly changing winter sun and wind conditions. Second, we applied both ordinary least squares and ordinal-logit regression to derive neutral temperature estimates, allowing a robustness check across model assumptions. Third, the use of 2,000-fold bootstrap confidence intervals provides an empirical measure of uncertainty without assuming normality or homoscedasticity, which is an important advantage given the nonlinear and individualised nature of thermal perception. Finally, our adoption of binned TSV-temperature regressions (in 0.5 °C increments) not only smoothed vote noise but allowed for intuitive visualisation of thermal sensitivity and model fit (R²) across sub-groups. Together, these methods offer a replicable framework for future field studies seeking to quantify subtle thermal shifts under mild winter conditions, and they align with recent calls for greater statistical rigour and cross-study harmonisation in outdoor thermal comfort research (Johansson et al. 2014; Potchter et al. 2018).

Despite the study’s large sample size and methodological robustness, several limitations should be acknowledged. First, our data reflect a single seasonal window (December-March) in one humid, subtropical city. While this timeframe captures a range of mild winter conditions, it does not permit comparison with transitional or summer seasons, which would allow for full seasonal comfort modelling or adaptation analysis. Second, the sampling strategy focused on current park users, not on non-users who may have been deterred by cold or wind. Future work could incorporate intercept surveys in adjacent indoor or semi-enclosed environments to understand environmental thresholds that inhibit outdoor participation. Third, while we included location and vegetation type, we did not explicitly quantify urban geometry (e.g., SVF, building height-width ratios). Incorporating fisheye photography or UAV-derived models could strengthen the causal link between form and thermal perception. Fourth, thermal physiology was not directly measured as variables such as skin temperature, heart rate variability, or sweat rate could provide a more mechanistic interpretation of age and sex differences. Lastly, this study was conducted in a high-density Asian city; although the findings may be transferable to cities with similar climates and demographics, future research in other cultural and urban contexts is needed to validate comfort ranges and preferences across subtropical regions.

## Conclusions

This study offers one of the most comprehensive winter-season outdoor thermal comfort assessments to date in a humid-subtropical Asian metropolis. Drawing on 611 paired questionnaire and microclimatic records collected across four urban parks in Hong Kong, we identified key patterns in neutral thermal conditions, demographic variability, and the influence of spatial context. Our results show that the neutral UTCI lies between 20 and 21 °C, with similar neutrality for air temperature (≈ 20.5 °C) and a slightly higher range for PET (≈ 23–24 °C). These values were consistent across regression techniques and remained stable under multiple robustness checks, including bootstrapping, model variation (OLS vs. ordinal-logit), and meteorological trimming. The strength of these results stems from our application of binned regression analysis and confidence intervals, which produced interpretable and statistically reliable benchmarks for urban design in the cool season.

Importantly, we found that thermal perception is not homogeneous. Age emerged as the most significant demographic variable: respondents over 75 years old exhibited neutral temperature 2–3 °C higher than younger adults, alongside flatter TSV-temperature gradients. These findings are consistent with prior physiological and behavioural studies and underscore the need to provide warmer microclimatic conditions for elderly park users, such as sunlit seating areas and wind-sheltered niches. Sex-based differences were more subtle: women displayed greater thermal sensitivity, evidenced by steeper regression slopes, though neutral points remained within overlapping confidence bands. This suggests that fine-grained design features, such as surface material selection or wind buffers, may be more perceptible to female users even if target conditions remain shared across sexes.

Spatial context also shaped comfort responses. Visitors to district parks with greater sky-view and vegetative openness exhibited warmer neutral UTCI and PET values than those in street parks, where air temperature played a more dominant perceptual role. These findings validate the idea of thermal zoning within parks so as to design for a spectrum of conditions that accommodate active and sedentary users, as well as younger and older cohorts. Design guidelines should aim to deliver baseline UTCI of 20–21 °C in general seating zones and offer + 2–3 °C pockets in locations intended for vulnerable populations. Overall, our findings provide evidence-based guidance for climate-responsive park design in mild-winter subtropical cities, especially in the context of ageing populations. Future work should explore other seasonal windows, integrate physiological monitoring, and test the scalability of these findings across diverse urban morphologies.

## Supplementary Information

Below is the link to the electronic supplementary material.

Supplementary Material 1 (DOCX 16.4 KB)

Supplementary Material 2 (DOCX 15.8 KB)

## Data Availability

Data will be made available upon request.
