# Supplementary material for: Designing for mild winters: evidence-based thermal comfort benchmarks from urban parks in a sub-tropical city
Source: Int J Biometeorol. 2026 Apr 7;70(4):116. doi: 10.1007/s00484-026-03190-9 (PMC13056736; doi:10.1007/s00484-026-03190-9)
Supplement: Supplementary file 1 — Supplementary Material 1 (DOCX 16.4 KB) [file 484_2026_3190_MOESM1_ESM.docx]

**Table S1. Clothing checklist items and corresponding garment insulation values (clo), based on standard reference tables and consistent with the garment dictionaries implemented in pythermalcomfort.**

| **Clothing item (as in questionnaire)** | **Standard reference item (closest match)** | **clo value (typical)** | **Notes (layer / season)** |
| --- | --- | --- | --- |
| T-shirt / short-sleeve shirt | T-shirt (0.08) or  short-sleeve dress shirt (0.19) | 0.08-0.19 | Base layer; select “T-shirt” if knit tee was reported, select “short-sleeve dress shirt” if woven shirt |
| Long-sleeve shirt | Long-sleeve dress shirt | 0.25 | Base layer |
| Sweater / knitwear | Long-sleeve sweatshirt or long-sleeve flannel shirt | 0.34 | Mid layer (warm knit or flannel equivalent) |
| Jacket (light) | Single-breasted coat (thin) | 0.36 | Outer layer, lightweight |
| Coat (heavy) | Single-breasted coat (thick) | 0.44 | Outer layer, heavyweight winter coat |
| Trousers | Straight trousers (thin) or straight trousers (thick) | 0.15-0.24 | Lower body; choose thin vs thick according to reported fabric/seasonal use |
| Jeans | Straight trousers (thick) (proxy) | 0.24 | Use as a conservative proxy if jeans were not separately coded |
| Shorts / skirt | Walking shorts (0.08) or skirt thin (0.14) or skirt thick (0.23) | 0.08-0.23 | Select by garment type and thickness |
| Socks | Ankle-length athletic socks (0.02) or calf-length socks (0.03) | 0.02-0.03 | Footwear layer; select length that matches checklist |
