# Supplementary material for: Designing for mild winters: evidence-based thermal comfort benchmarks from urban parks in a sub-tropical city
Source: Int J Biometeorol. 2026 Apr 7;70(4):116. doi: 10.1007/s00484-026-03190-9 (PMC13056736; doi:10.1007/s00484-026-03190-9)
Supplement: Supplementary file 2 — Supplementary Material 2 (DOCX 15.8 KB) [file 484_2026_3190_MOESM2_ESM.docx]

**Table S2. Activity categories to metabolic rate (met) used in PET/UTCI calculations, based on standard ‘typical task’ met values consistent with pythermalcomfort reference tables.**

| **Activity category (questionnaire)** | **Example activities** | **Metabolic rate (met)** | **Source / rationale** |
| --- | --- | --- | --- |
| Sitting / resting | seated, chatting | 1 | “Seated, quiet” typical value |
| Standing | standing, waiting | 1.2 | “Standing, relaxed” typical value |
| Walking / strolling | slow walking | 2 | “Walking 2 mph” typical value |
| Light exercise | tai chi, stretching | 2.6 | Use a light-to-moderate movement proxy consistent with “Walking 3 mph” in typical-task tables; if you prefer a more conservative upper bound, report a sensitivity check using 3.5 met (“calisthenics”). |
